# Incidence and risk factors for postintensive care syndrome in a cohort of critically ill patients

**DOI:** 10.5935/0103-507X.20220224-en

**Published:** 2022

**Authors:** Julia Tejero-Aranguren, Raimundo García-del Moral Martin, Maria Eugenia Poyatos-Aguilera, Ildaura Morales-Galindo, Angel Cobos-Vargas, Manuel Colmenero

**Affiliations:** 1 Intensive Care Department, Hospital Universitario Clínico San Cecilio, POD Medicina Clínica y Salud Pública, Universidad de Granada - Granada, Spain.

**Keywords:** Intensive care, Critically illness, Postintensive care syndrome, Incidence, Risk factors

## Abstract

**Objective:**

To determine the incidence of postintensive care syndrome in a cohort of critically ill patients admitted to the intensive care unit and to identify risk factors related to its development in the physical, cognitive and mental health areas.

**Methods:**

This was a prospective observational cohort study developed in the intensive care unit of a university hospital. Patients with intensive care unit stays equal to or longer than one week and the need for mechanical ventilation for more than 3 days, shock or *delirium* were included in the study. Demographic variables, reasons for admission, diagnoses, sedation, type of mechanical ventilation used, complications and length of stay were recorded. A univariate analysis was performed to identify risk factors related to postintensive care syndrome. The scales used for the assessment of the different spheres were Barthel, Pfeiffer, Hospital Anxiety and Depression Scale and Impact of Event Scale-6. The main variables of interest were postintensive care syndrome incidence overall and by domains. Risk factors were examined in each of the health domains (physical, cognitive and mental health).

**Results:**

Eighty-seven patients were included. The mean Acute Physiology and Chronic Health Evaluation II score was 16.5. The mean number of intensive care unit days was 17. The incidence of global postintensive care syndrome was 56.3% (n = 49, 95%CI 45.8 - 66.2%). The incidence of postintensive care syndrome in each of the spheres was 32.1% (physical), 11.5% (cognitive), and 36.6% (mental health).

**Conclusions:**

The incidence of postintensive care syndrome is 56.3%. The mental health sphere is the most frequently involved. The risk factors are different depending on the area considered.

## INTRODUCTION

Technological advances in intensive care units (ICUs) in recent years have improved survival rates, but a large number of patients present alterations derived from prolonged admission to the ICU. Postintensive care syndrome (PICS) is a term used to describe new or worsening multidimensional impairments in physical, cognitive and mental health arising from critical illness and persisting beyond hospital discharge. All of these impairments, whether in the physical sphere, cognitive sphere or mental health sphere, are included within the syndrome, which affects up to 50% of patients who survive admission to the ICU.^(1,2)^

Experience in the follow-up and treatment of this type of patient is extensive in countries such as England and the United States, where there are specific rehabilitation centers for patients who have survived a critical illness.^(3)^ In Spain, measures aimed at early diagnosis of the syndrome and its treatment as well as interdisciplinary collaboration for the development of a set of preventive measures to minimize its impact have recently begun to be implemented. There is currently a national working group called ITACA^(4)^ in which multiple centers collaborate in the study of PICS. Despite the publication of a post-ICU follow-up protocol, some data on the development of mental health disorders^(5)^ and the impact of PICS on family members,^(6)^ there are no data on the incidence and risk factors for PICS in Spain.

The identification of risk factors for the development of this syndrome has been performed through registries and retrospective studies in patients with a specific pathology (acute respiratory distress syndrome - ARDS^(7,8)^ or sepsis^(9,10)^). There are few studies in a heterogeneous population of critically ill patients, as we usually see in clinical practice. Different risk factors have been identified depending on the area of health analyzed, but which of these may be potentially modifiable and the strategies to be employed remain to be clarified.

The aim of this study was to determine the incidence of PICS in a cohort of critically ill patients admitted to the ICU and to identify risk factors related to its development in the physical, cognitive and mental health spheres.

## METHODS

This prospective cohort study was performed in a university hospital with 20 ICU beds and an average of 1,200 admissions per year from January 1, 2018, to January 1, 2020. All patients with ICU stays equal to or longer than one week and at least one of the following criteria were included: need for mechanical ventilation (MV) for more than 3 days, shock and/or *delirium* in the ICU. Patients with a high degree of functional dependence on admission to the ICU (Barthel Index score between 21 and 60 points) or a previous diagnosis of cognitive impairment were excluded.

### Follow-up protocol in postintensive care syndrome consultation

The assessment was performed 3 months after hospital discharge. The scales used for the assessment of the different spheres were the Barthel scale (physical), Pfeiffer test (cognitive), Hospital Anxiety and Depression Scale (HADS) and Impact of Event Scale-6 (IES-6) (mental health). The questionnaires were administered by two of the investigators, each of whom had demonstrated competence in performing the questionnaires after a mock interview with the principal investigator.

The variables were demographic data and the reason for admission to the ICU; ICU admission assessment scales (both severity and functional and cognitive assessment); development of shock during the ICU stay; days of noninvasive mechanical ventilation (NIV) or high-flow nasal therapy; days of invasive MV; need for tracheostomy; development of ARDS and its degree according to the Berlin conference criteria from 2012; days of deep sedation measured as the Richmond Agitation-Sedation Scale ≥ -4; presence or absence of *delirium* defined as positive by the Confusion Assessment Method in ICU (CAM-ICU) and duration thereof; presence or absence of polyneuropathy of the critically ill patient at ICU discharge defined as a score on the Medical Research Council scale of muscle strength less than 48;^(11)^ presence or absence of dysphagia at ICU discharge; days of ICU admission; and days of hospital admission after ICU discharge. high-flow nasal cannula oxygen.

### Definition of postintensive care syndrome

Postintensive care syndrome was considered to be the appearance of alterations in any of the three spheres. Physical alteration was defined as deterioration in one category on the Barthel dependency scale with respect to ICU admission; cognitive alteration was defined as a score higher than 3 points on the Pfeiffer test; and mental health alteration was defined as a score higher than 11 on the HADS test and/or 1.75 on the IES-6 score for posttraumatic stress disorder.

### Statistical analysis

As this was a descriptive study with the aim of generating working hypotheses, the sample size was one of convenience. The results are expressed according to the type of variable. Continuous variables are expressed as the means, medians and interquartile range (IQR). Categorical variables are expressed as absolute values and percentages. Univariate analysis of the continuous variables was performed with Student’s test for age (fulfilling the hypothesis of normality) and the Wilcoxon test for the time variables (length of stay, days of sedation and invasive MV) as it was not possible to assume normality of these variables. Binary logistic regression was used for the univariate analysis of the qualitative variables and for the estimation of the odds ratio (OR). Differences with p < 0.05 were considered significant. The data were anonymized for analysis. R software version 4.0.3 (R Foundation for Statistical Computing Platform, Vienna, Austria) r-commander 2.6-2 package was used.

The project was approved by the center’s Research Ethics Committee, and consent to participate was requested from patients and family members.

## RESULTS

**Figure 1:**
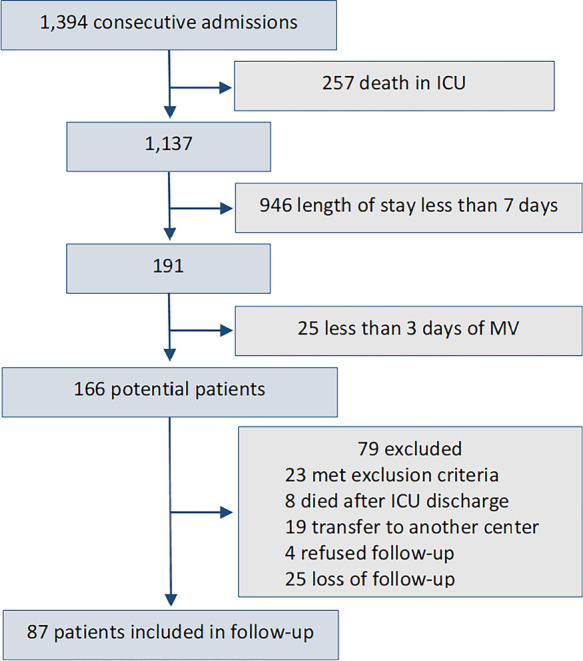
Patients included in the study.

During the study period, 1,394 patients were admitted to the ICU. The patient inclusion flow chart is shown in figure 1. Eighty-seven patients were included, 48 of whom were male (55.2%). The mean age was 58.1 years (standard deviation - SD 13.8). The median ICU stay was 17 days (IQR 22.75), with a maximum of 84 days. The characteristics of the cohort are shown in table 1.

**Table 1:** Characteristics of the cohort of patients included in the follow-up

	Median	Range	Mean	SD
Age (years)	60	17 - 86	58.1	13.8
APACHE II (points)	13	3 - 42	16.4	10.5
MV (days)	9	0 - 21	16.2	17
ICU LOS (days)	17	7 - 33	24.2	19.1
Post-ICU Hospital LOS (days)	13	1 - 22	18.1	15.3

SD - standard deviation; APACHE II - Acute Physiology and Chronic Health Evaluation II; MV - mechanical ventilation ICU - intensive care unit; LOS - length of stay.

ICU - intensive care unit; MV - mechanical ventilation.

The mean Acute Physiology and Chronic Health Evaluation II (APACHE II) score at admission was 16.4 (SD 10.5). Tracheostomy was performed in 35 patients (40.2%). From the group of patients active at admission (n = 52), 25 patients (48%) had returned to work within three months of discharge.

**Figure 2:**
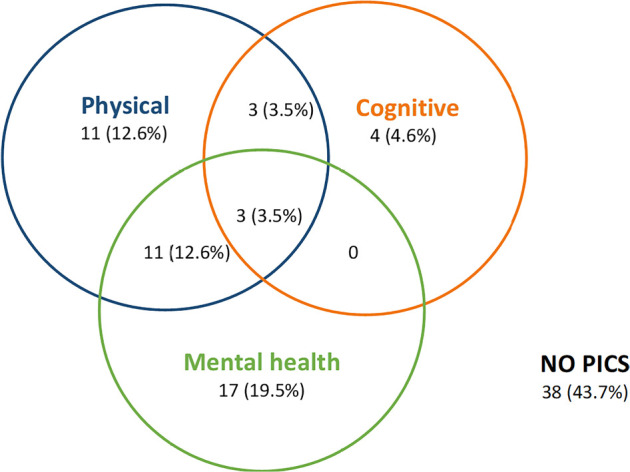
The incidence of postintensive care syndrome.

The incidence of PICS (including all three spheres) was 56.3% (n = 49, 95% confidence interval (95%CI) 45.8% - 66.2%), as shown in figure 2. In the univariate analysis, the different risk factors for the development of PICS are shown in table 2.

**Table 2:** Risk factors for the development of global postintensive care syndrome

Variable	PICS		OR	p value
	No	Yes		
Age (years)	58.6	57.8	0.9	0.8
Female	14 (36)	25 (64)	0.56	0.18
Male	24 (50)	24 (50)		
APACHE II	12.5	13	1.004	0.81
No ARDS	15 (41.7)	21 (58.3)	0.86	0.75
ARDS	23 (45)	28 (55)		
No septic shock	18 (42)	25 (58)	0.96	0.86
Septic shock	20 (45.5)	24 (54.5)		
No CIP	29 (52.7)	26 (47.3)	2.85	0.02
CIP (at ICU discharge)	9 (28)	23 (72)		
No *delirium*	21 (52.5)	19 (47.5)	1.95	0.12
*Delirium*	17 (36.2)	30 (63.8)		
ICU LOS (days)	12	30	1.04	0.07

PICS - post-intensive care syndrome; OR - odds ratio; APACHE II - Acute Physiology and Chronic Health Evaluation II; ARDS - acute respiratory distress syndrome; CIP - critical illness polyneuropathy; ICU - intensive care unit; LOS - length of stay. The results are expressed as the mean, n (%) or median.

PICS - postintensive care syndrome.

In the physical sphere, 28 patients (32.2%) met the criteria for PICS. The variables related to the development of PICS (increased risk) were age, presence of polyneuropathy at ICU discharge and time variables (ICU stay, days of sedation, days of MV and post-ICU hospital stay).

In the cognitive sphere, ten patients (11.5%) presented PICS. Factors associated with an increased risk of PICS are severity measured by the APACHE II scale and days of hospital stay after ICU discharge.

In the mental health sphere and according to the different criteria used, the incidence was as follows: IES score > 1.75: 22 (25.3%) meet criteria for posttraumatic stress disorder and HADS scale score > 11 points: 26 (29.8%)

Considering the occurrence of PICS in mental health as the occurrence of any of the following items, the incidence of PICS-mental health was 31 (34.2%). We did not find any factor associated with the development of alterations in the mental health sphere (Table 2). The use of high-flow nasal cannula oxygen therapy (HFNC) or NIV in the ICU was not a risk factor for the development of PICS in mental health (30.4% *versus* 40.4%, p = 0.31, OR for HFNC/NIV use = 1.58) or for the development of posttraumatic stress disorder (20.5 *versus* 30.4; p = 0.29, OR for HFNC/NIV use = 1.69) in our cohort of patients.

## DISCUSSION

The incidence of PICS in our cohort of critically ill patients was 56.3%, which indicates that one out of two patients will be affected by this disorder. Alterations in the mental health sphere are the most frequently involved, closely followed by physical alterations, with cognitive disorders being the least frequent. The risk factors are different depending on the sphere considered. Physical involvement is conditioned by age, the presence of polyneuropathy at ICU discharge and the time variables of stay, sedation and invasive MV. Cognitive impairment is conditioned by severity at admission and hospital stay. We did not identify potential risk factors for mental health impairment.

The incidence of PICS is different depending on the time after discharge and the characteristics of the patient population. It has been studied over a wide time range from 3 to 12 months after hospital discharge, ranging from 64% at 3 months to 56% at one year, with the coexistence of alterations in the different spheres being common.^(1,12)^ Publications on PICS have focused on specific pathologies and in the context of multicenter studies with other objectives, the most frequently studied being ARDS^(7,8)^ and sepsis.^(9,10)^ Our study encompasses a cohort of critically ill patients with different reasons for admission and in the routine clinical practice of an ICU. The incidence in our cohort of patients is similar to that described by other authors in medical ICUs. Thus, Maley et al.,^(1)^ based on 43 patients with more than 2 days of stay, found an incidence of PICS of 56%, and Marra et al.^(13)^ described an incidence of PICS of 64% 3 months after hospital discharge in the follow-up of 406 patients with respiratory failure or shock.

There is great heterogeneity in the instruments used for the assessment of physical PICS. Recently, the Society of Critical Care Medicine^(14)^ performed a systematic review to identify the risk factors associated with PICS as well as the best tools to identify it. The 6-minute walk test is recommended,^(15,16)^ with a low grade of recommendation. We opted for the Barthel scale^(17)^ because of its simplicity, objectivity and the possibility of applying it by telephone. In addition, this scale is widely used in the assessment at admission and discharge from the ICU and the hospital, which allows us to compare the patient’s previous situation with the situation at the follow-up visit.^(16)^

The variables related to physical PICS are age, the presence of polyneuropathy at ICU discharge and the time-dependent variables: the length (in days) of sedation and MV, ICU stay and hospital stay. These results are in agreement with what has been described thus far in the literature. With respect to age as a risk factor, other authors have already described a lower degree of recovery in patients over 70 years of age after admission to the ICU, with worse scores in physical tests, such as the Medical Research Council dyspnea scale and 6-minute walk test, and a greater degree of functional dependence.^(8,18)^ In the RECOVER study,^(3)^ age and days of ICU admission were postulated to be potent modulators of subsequent physical deterioration. The development of polyneuropathy in critically ill patients affects approximately 40% of ICU patients;^(19,20)^ is usually accompanied by respiratory muscle involvement in 80% of cases, is associated with a greater need for days of MV, and consequently is associated with a greater number of days of MV, which translates into more days of ICU admission.^(21-23)^ Finally, the impact of days of deep sedation on our results agrees with Jackson et al.,^(24)^ who, studying the impact of sedation protocols on post-ICU recovery, found significant differences in functional status at 1 year between the two study groups, the first with daily sedation interruption protocol and the second with a usual sedation protocol (64% *versus* 87%; p = 0.05).

The incidence of cognitive impairment was lower than that described by other authors,^(25)^ who placed it between 20 and 40%. The scales used for the evaluation of cognitive impairment are diverse, including the MoCa test^(26)^ (Montreal Cognitive Assessment) and the Pfeiffer test.^(27)^ In our case, the Pfeiffer test may have underdiagnosed cognitive impairment as it is a screening test, and it has reported lower incidences than other more complex neuropsychological tests, such as the MoCa test.^(27)^ The exclusion of patients with previous cognitive impairment^(1)^ and the difference in patient profile may also have influenced the lower incidence compared with that described in the literature.^(28)^ The factors associated with cognitive impairment in our sample are severity measured by the APACHE II scale and days of hospital stay. The severity of critical illness has already been described as a risk factor; it is related to the presence of multiorgan failure, endothelial damage and thrombotic and inflammatory events that are postulated to be behind the etiopathogenesis of brain damage causing cognitive impairment.^(24)^ We found no association between *delirium* and cognitive impairment at 3 months post-ICU, despite *delirium* being a factor frequently associated with post-ICU cognitive impairment.^(19,29,30)^ The small number of patients with cognitive impairment in our cohort (n = 11) may have conditioned the significance of this variable.

The incidence of mental health alterations was 36.6% (31 patients). Approximately one-third of ICU survivors present signs of depression, and one in four patients present symptoms compatible with posttraumatic stress syndrome.^(12)^ We did not identify significant risk factors. The scales used for assessment were those recommended by scientific societies^(13)^ and the best validated studies on the subject.^(31,32)^ We believe that this may be because alterations in the mental health sphere are strongly influenced by previous personality alterations or other factors after admission to the ICU, such as family and/or social support and the ability to return to work.

There has been speculation about the role of NIV and HFNC in the development of posttraumatic stress disorder,^(33)^ with patients being awake and alert during their stay in the ICU, which can generate high levels of stress and perception of the severity of patients in the environment. Although there are differences of up to 10% for both the diagnosis of PICS in mental health and for the development of posttraumatic stress disorder, there is no evidence of a significant difference between the two.

Our study has the following limitations. As it is a single-center study with a limited number of patients, multivariate analysis could not be performed. The findings should be considered preliminary. The scales used have allowed their use even when a face-to-face visit to the consultation was not possible, as they could be made by telephone. However, some of these scales are not those currently recommended by the Society of Critical Care Medicine, as the recommendations were published after the start of our study.

The study has its strengths; it is a portrait of the reality of a medical ICU with a cohort of patients with multiple pathologies that groups together the risk factors most frequently described in the current literature and that does not focus on only one area (physical, cognitive or mental health) or a specific pathology.

## CONCLUSION

The incidence of postintensive care syndrome affects one out of every two patients who survive a critical illness. This high percentage should induce us to follow up this type of patient at intensive care unit discharge to identify those who could benefit from specific and specialized treatment. On the other hand, the risk factors for the three spheres of postintensive care syndrome are different. Some of them are not modifiable (age and severity), but others can be (sedation and mechanical ventilation times, and polyneuropathy of the critically ill patient); therefore, strategies adapted to specific objectives should be used for their prevention.
